# SARS-CoV-2-specific CD4^+^ T cell longevity correlates with Th17-like phenotype

**DOI:** 10.1016/j.isci.2022.104959

**Published:** 2022-08-17

**Authors:** Kazutaka Terahara, Takashi Sato, Yu Adachi, Keisuke Tonouchi, Taishi Onodera, Saya Moriyama, Lin Sun, Tomohiro Takano, Ayae Nishiyama, Ai Kawana-Tachikawa, Tetsuro Matano, Takayuki Matsumura, Masaharu Shinkai, Masanori Isogawa, Yoshimasa Takahashi

**Affiliations:** 1 Research Center for Drug and Vaccine Development, National Institute of Infectious Diseases, Tokyo 162-8640, Japan; 2 Tokyo Shinagawa Hospital; Tokyo, 140-8522, Japan; 3 Department of Life Science and Medical Bioscience, Waseda University, Tokyo, 162-8480, Japan; 4 AIDS Research Center, National Institute of Infectious Diseases, Tokyo, 162-8640, Japan

**Keywords:** Health sciences, Medicine, Immunology, Virology, Biological sciences

## Abstract

Determinants of memory T cell longevity following severe acute respiratory syndrome coronavirus 2 (SARS-CoV-2) infection remain unknown. In addition, phenotypes associated with memory T cell longevity, antibody titers, and disease severity are incompletely understood. Here, we longitudinally analyzed SARS-CoV-2-specific T cell and antibody responses of a unique cohort with similar numbers of mild, moderate, and severe coronavirus disease 2019 cases. The half-lives of CD4^+^ and CD8^+^ T cells were longer than those of antibody titers and showed no clear correlation with disease severity. When CD4^+^ T cells were divided into Th1-, Th2-, Th17-, and Tfh-like subsets, the Th17-like subset showed a longer half-life than other subsets, indicating that Th17-like cells are most closely correlated with T cell longevity. In contrast, Th2- and Tfh-like T cells were more closely correlated with antibody titers than other subsets. These results suggest that distinct CD4^+^ T cell subsets are associated with longevity and antibody responses.

## Highlights

• Th17-like CD4^+^ T cells showed a longer half-life than other CD4^+^ T cell subsets
• Anti-RBD-IgG titers were associated with Th2- and Tfh-like CD4 T cells
• CD45RA+CD8^+^ T cells were correlated with disease severity during the early phase

## Graphical abstract

**Figure:**
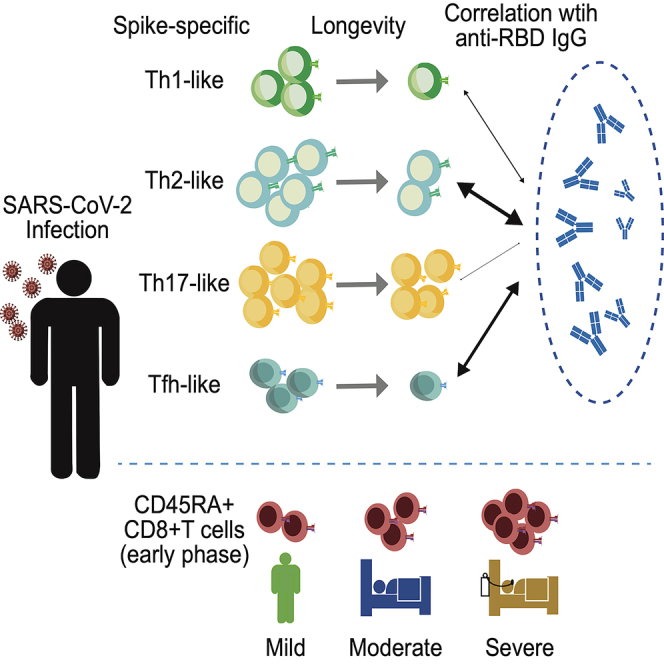

## Teaser

Health sciences; Medicine; Immunology; Virology; Biological sciences; Immunology; Virology.

## Introduction

The novel severe acute respiratory syndrome coronavirus 2 (SARS-CoV-2) causes coronavirus disease 2019 (COVID-19), which is an ongoing pandemic that has killed more than 5 million people worldwide as of December 2021. SARS-CoV-2 infection triggers virus-specific cellular and humoral immune responses. While the coordination of both arms of adaptive immunity is deemed critical for resolving COVID-19 (Bert et al., 2020; Moderbacher et al., 2020; Sekine et al., 2020; Sette and Crotty, 2021), accumulating evidence suggests that the kinetics and magnitude of SARS-CoV-2-specific T cells and antibody responses do not necessarily correlate with each other. For example, SARS-CoV-2-specific antibody titers usually correlate with disease severity in COVID-19 patients, i.e., severe COVID-19 patients possess higher antibody titers than mild or moderate COVID-19 cases (Chen et al., 2020; Garcia-Beltran et al., 2021; Piccoli et al., 2020). In contrast, SARS-CoV-2 specific CD4^+^ and CD8^+^ T cell responses are associated with reduced disease severity (Moderbacher et al., 2020) and the appearance of SARS-CoV-2-specific T cell responses is delayed and reduced in severe COVID-19 cases (Tan et al., 2021). In addition, serum antibody titers are poorly predictive of the presence of SARS-CoV-2-specific CD4^+^ T cells (Dan et al., 2021). It is currently unknown whether the relationship between disease severity, T cell responses, and antibody titers changes over time. Longitudinal analysis of SARS-CoV-2-specific immune responses at multiple time points in each case is essential for understanding the factors that influence T cell longevity as well as the basis of disparity between the T cell and antibody response. Identifying determinants for T cell longevity is urgently needed due to rapidly emerging variants of concern (VOCs) that can evade antibody neutralization (Cele et al., 2021; Karim and Karim, 2021; Nemet et al., 2021).

T cells, particularly CD4^+^ T cells, are versatile and able to differentiate into distinct subsets, such as Th1, Th2, Th17, and so on, each of which expresses a unique chemokine receptor (DuPage and Bluestone, 2016). Information on T cell subsets in COVID-19 is still limited. Tfh cells are required for most Immunoglobulin G (IgG) responses and potently neutralizing antibodies and distinguished by CXCR5 expression. Puzzlingly, reduced disease severity was correlated with circulating CXCR5^+^ SARS-CoV-2-specific CD4^+^ T cell frequencies but not with antibody titers (Long et al., 2020; Moderbacher et al., 2020; Robbiani et al., 2020). Thus far, the association between circulating Tfh (cTfh) and antibody titers remains inconclusive. Th1 and Th17 are often characterized by CXCR3 (Ward et al., 1998) and CCR6 (Singh et al., 2008), respectively, and CXCR3^+^ and CCR6^+^ SARS-CoV-2-specific CD4^+^ T cells were both detected during acute COVID-19 (Juno et al., 2020; Moderbacher et al., 2020). However, their durability and role in pathogenesis have not been adequately addressed. SARS-CoV-2-specific CD8^+^ T cells are also detectable in acute COVID-19 cases, albeit less consistently than CD4^+^ T cells, and their presence was also correlated with a better outcome of COVID-19 (Bert et al., 2021; Moderbacher et al., 2020; Sekine et al., 2020; Tan et al., 2021). Whether any particular CD8^+^ T cell subset contributes to disease severity remains unknown.

In this study, we analyzed the frequency and phenotypes of SARS-CoV-2-specific CD4^+^ and CD8^+^ T cell responses in peripheral blood mononuclear cells (PBMCs) of a unique cohort that consists of similar numbers of mild, moderate, and severe cases. Most subjects contributed blood samples four times for up to 15 months after disease onset, allowing us to determine the half-life of T cell responses and the correlation between T cell responses and antibody titers without being affected by subject-to-subject variations. The study identifies distinct CD4^+^ T cell subsets uniquely associated with T cell longevity, antibody titers.

## Results

### Study cohort

**Figure 1:**
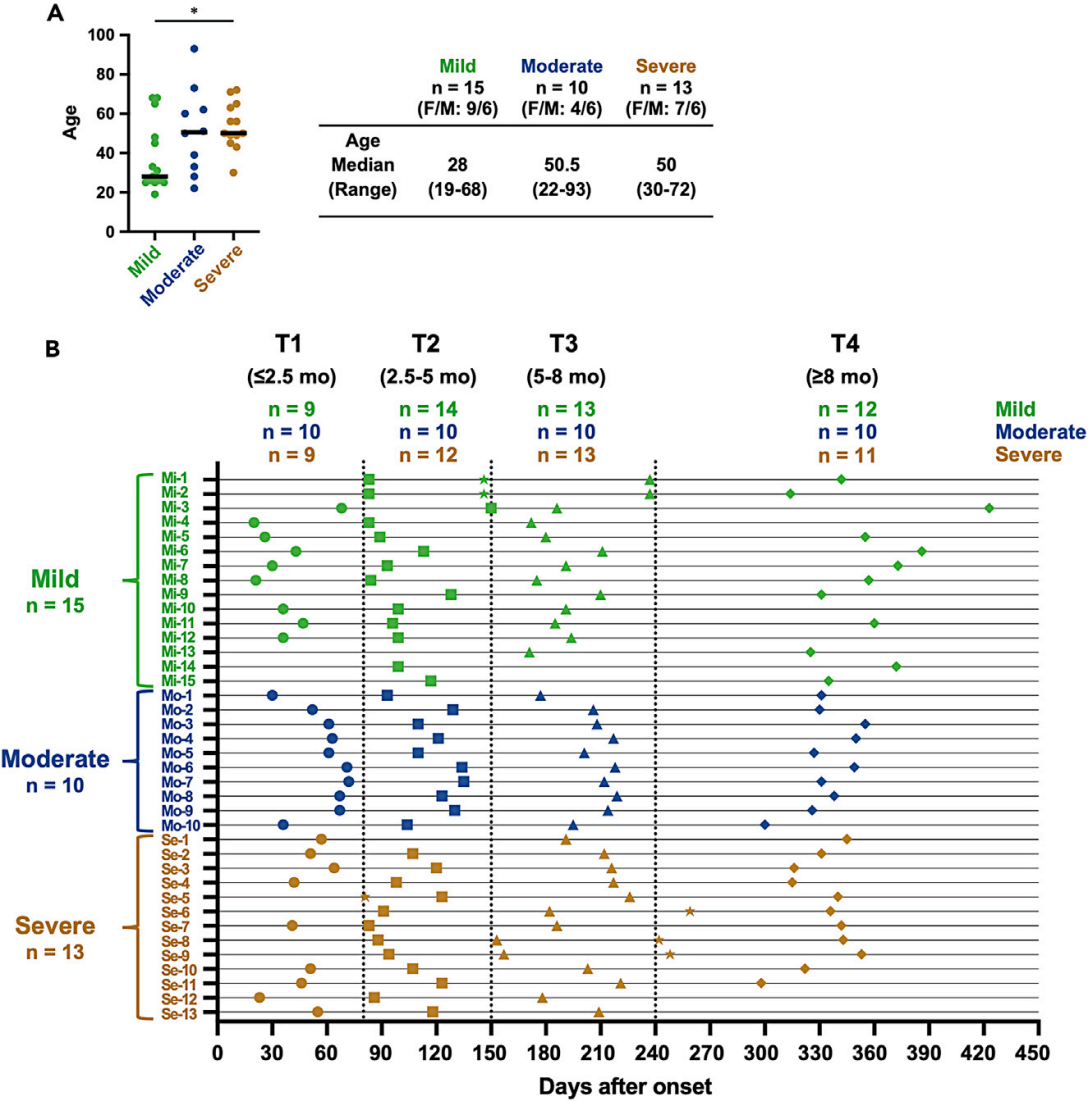
Timeline of sample collection for longitudinal analysis (A) The demographics and clinical characteristics of participants. Significant differences (∗p < 0.05) were determined by the Kruskal-Wallis test followed by the Dunn multiple comparison test. F: female, M: male. (B) Two or more samples were collected individually from a total of 38 convalescent individuals (mild: n = 15, moderate: n = 10, and severe: n = 13) during approximately 1 year after symptom onset. Time points were divided into four (T1 [circle symbol]: ≤2.5 months, T2 [square symbol]: 2.5–5 months, T3 [triangle symbol]: 5–8 months, and T4 [diamond symbol]: ≥8 months). If subjects provided samples twice in the same period, samples collected closer to the median date were chosen to represent the period. However, other samples (star symbol) were used for longitudinal analyses and included in correlation analyses using all samples.

Thirty-eight individuals with PCR-confirmed COVID-19 were enrolled in this study. None of them were vaccinated against COVID-19. The disease severity was evaluated according to the NIH severity of illness categories (available: https://www.covid19treatmentguidelines.nih.gov/) (2021). The cohort consists of 15 cases with mild, 10 with moderate, and 13 with severe COVID-19. The demographics and clinical characteristics of participants are summarized in Figure 1A. Subjects with mild COVID-19 were younger than moderate and severe cases, but statistical significance was only observed between mild and severe cases (mild vs. moderate: p = 0.1916, mild vs. severe: p = 0.0311). All subjects provided samples at least twice, and most subjects (28 of 38 subjects) contributed four times, over 14 months post symptom onset. Accordingly, we divided the convalescent period into 4 phases as follows: T1 (<2.5 months), T2 (2.5–5 months), T3 (5–8 months), and T4 (>8 months), and the number of samples from mild, moderate, and severe cases in each phase was indicated above the schema (Figure 1B).

### SARS-CoV-2-specific memory T cell responses are sustained 1 year after infection

**Figure 2:**
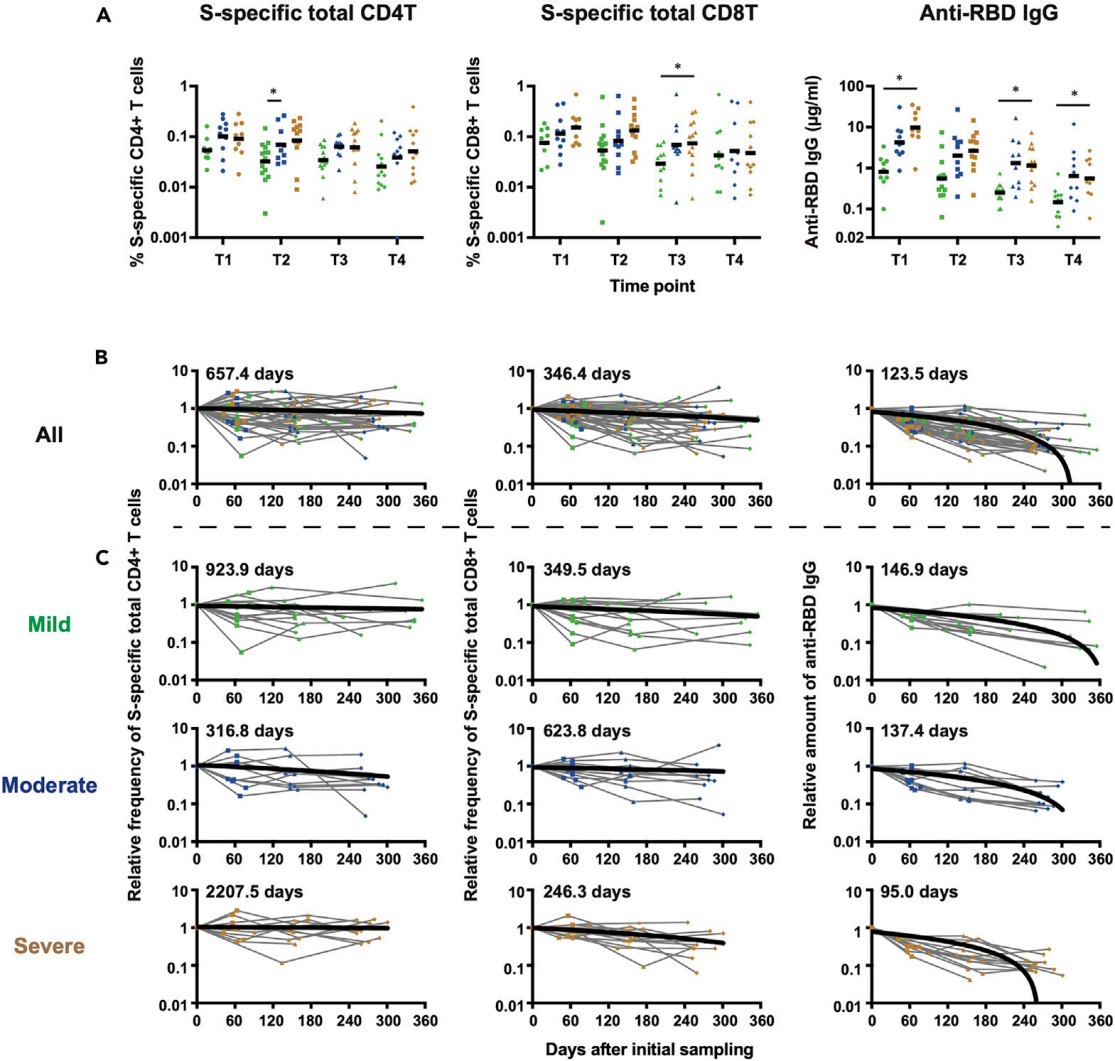
Kinetics of S-specific T-cell frequencies and anti-RBD IgG titers by severity (A) S-specific CD4^+^ T cell frequencies in each time point. Significant differences (∗p < 0.05) were determined by the mixed effects model followed by Tukey multiple comparison test. (B and C) The half-life for S-specific T-cell frequencies and anti-RBD IgG titers in all subjects (B) and subjects divided into three groups based on disease severity (C) was calculated by linear regression analysis, in which the initial sampling day was set as day 0.

SARS-CoV-2-specific T cell responses were longitudinally analyzed by activation-induced marker assay using overlapping peptide pools spanning the spike (S) and nucleocapsid (N) proteins as described in the STAR METHODS and Figure S1. Immunoglobulin G titers against spike receptor-binding domain (RBD) and N protein were also measured by enzyme-linked immunosorbent assay (ELISA). S-specific and N-specific CD4^+^ and CD8^+^ T cell responses were induced in virtually all subjects and similarly maintained for approximately 1 year (Figure 2A and S2A). When the T cell responses were compared among three groups based on disease severity, no clear difference was observed in each time point, although the frequency of antigen-specific T cells tended to be lower in the mild cases. In contrast to T cell responses, anti-RBD IgG titers were significantly lower in mild cases, especially when compared with those in severe cases (Figure 2A). Anti-N IgG titers also tended to be lower in the mild cases, but no significant difference was observed among the three (mild, moderate, and severe) groups (Figure S2A). As reported elsewhere (Dan et al., 2021; Rodda et al., 2021; Wheatley et al., 2021), antibody titers appeared to decay more rapidly than T cell responses.

We then analyzed the half-life of SARS-CoV-2-specific T cell responses and antibody titers. So far, in many studies, the half-life was mostly calculated based on raw T cell frequencies (Dan et al., 2021; Jung et al., 2021), which can be profoundly influenced by subject-to-subject variations. To estimate the half-life more precisely, we determined fold changes of T cell frequencies and antibody titers relative to the value of initial sampling for each subject. As shown in Figure 2B, the half-life of S-specific CD4^+^ T cells and CD8^+^ T cells were approximately 657.4 and 346.4 days, respectively, indicating that CD4^+^ T cell responses were more stable than CD8^+^ T cell responses. A similar tendency was observed with N-specific CD4^+^ and CD8^+^ T cells (Figure S2B), although the half-life of N-specific CD8^+^ T cells may not be accurate due to a significant sample-to-sample variation. When samples were stratified by disease severity, we did not see clear correlation between T cell longevity and disease severity. As expected, the half-lives of antibodies (123.5 days for anti-RBD IgG, 128.9 days for anti-N IgG) were shorter than those of T cells.

### CCR6+ Th17-like T cells are more durable than other T cell subsets

**Figure 3:**
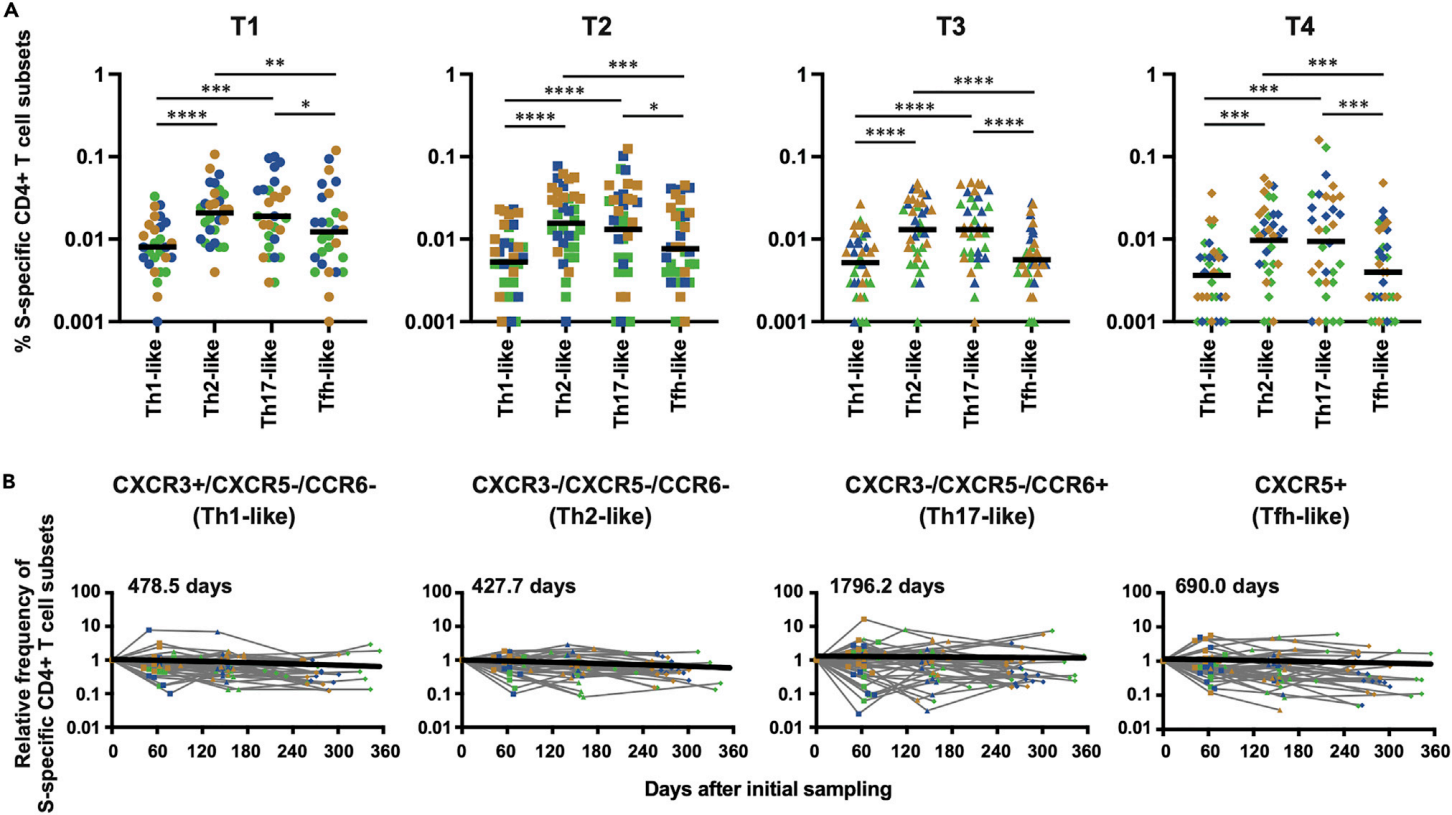
Dynamics of S-specific CD4^+^ T cell frequencies at subset levels (A) Comparison of S-specific cell frequencies between CD4^+^ T cell subsets at each time point. Significant differences (∗p < 0.05, ∗∗p < 0.01, ∗∗∗p < 0.001, ∗∗∗∗p < 0.0001) were determined by the Friedman test followed by the Dunn multiple comparison test. (B) Kinetics of S-specific CD4^+^ T cell frequencies at subset levels (CXCR3^+^ CXCR5^−^ CCR6^-^: Th1-like, CXCR3^−^ CXCR5^−^ CCR6^-^: Th2-like, CXCR3^−^ CXCR5^−^ CCR6^+^: Th17-like, and CXCR5^+^: Tfh-like). Half-life was calculated by linear regression analysis, in which the initial sampling day was set as day 0.

To further characterize SARS-CoV-2-specific T cell responses, we analyzed SARS-CoV-2-specific CD4^+^ T cells for the expression of various chemokine receptors, such as CXCR5, CXCR3, and CCR6, which are often used to determine T cell subsets (Kasatskaya et al., 2020; Long et al., 2021; Morita et al., 2011). Here, we defined CXCR3^+^/CXCR5^-^/CCR6^-^, CXCR3^-^/CXCR5^-^/CCR6^-^, CXCR3^-^/CXCR5^-^/CCR6^+^, and CXCR5^+^ CD4^+^ T cells as Th1-like, Th2-like, Th17-like, and Tfh-like cells, respectively (Figure S1). As shown in Figure 3A, S-specific Th2- and Th17-like cells were more frequent than Tfh- and Th1-like cells at every time point, suggesting that T cell subset hierarchy is maintained over an extended period. The hierarchy is also maintained when subjects are divided into three groups according to disease severity (Figure S3). We then calculated the half-life of each T cell subset as described above. As shown in Figure 3B, the half-life of Th1-, Th2-, Th17-, and Tfh-like cells were 478.5, 427.7, 1796.2, and 690.0 days, respectively, indicating that Th17-like cells are most closely associated with T cell longevity.

### T cell subsets differentially correlate with antibody responses

**Figure 4:**
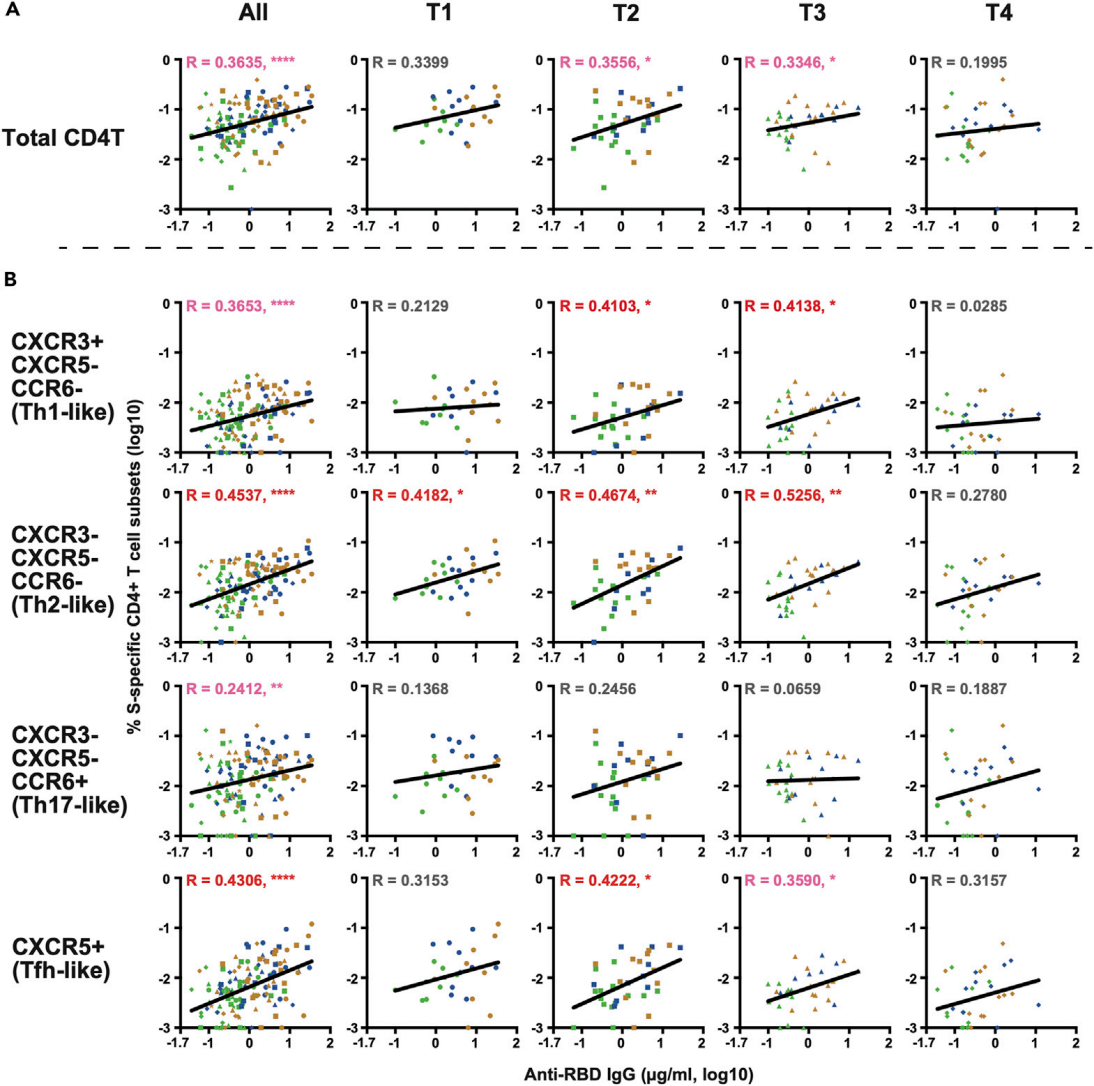
Correlation between S-specific CD4^+^ T cell frequencies and anti-RBD IgG titers by time points (A) Correlation between total S-specific CD4^+^ T cell frequencies and anti-RBD-IgG titers. (B) Correlation between individual S-specific CD4^+^ T cell subset and anti-RBD-IgG titers. The Spearman rank correlation coefficient was used for statistical analysis. Lines in the figures represent the best fit curve of simple linear regression analyses. Red and pink characters indicate R ≥ 0.4 and R < 0.4, respectively, with statistical significance (∗p < 0.05, ∗∗p < 0.01, ∗∗∗p < 0.001, ∗∗∗∗p < 0.0001).

Next, the correlation between SARS-CoV-2-specific T cell frequencies and antibody titers was examined at multiple time points. As shown in Figure 4A, S-specific CD4^+^ T cell frequencies only modestly correlated with anti-RBD IgG titers. Interestingly, the correlation tended to be stronger at T2 and T3 (i.e., between 2.5 and 8 months after onset) with statistical significance than at T1 (<2.5 months) or T4 (>8 months). We then examined the correlation between individual T cell subsets and anti-RBD IgG titers. As shown in Figure 4B, anti-RBD IgG titers were correlated most closely with the frequencies of Th2-like cells and least closely with those of Th17-like cells. At T4, none of the T cell subsets showed a significant correlation with anti-RBD IgG. N-specific CD4^+^ T cell frequencies showed virtually no correlation with anti-RBD IgG titers (Figure S4), raising the possibility that CD4^+^ T cells and B cells need to recognize the cognate antigen for antibody production. However, none of the N-specific T cell subsets correlated with anti-N IgG titers (Figure S5).

**Figure 5:**
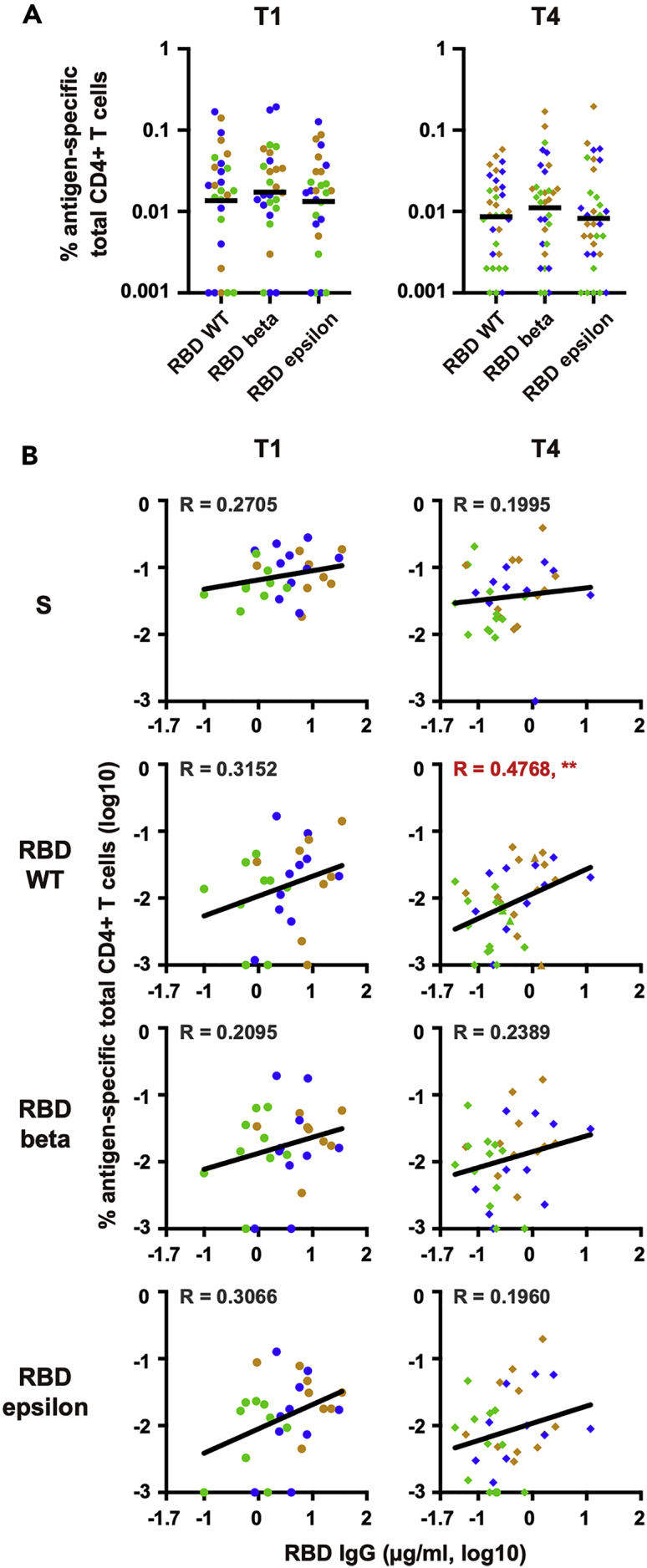
Profiling of RBD-specific CD4^+^ T cell responses (A) CD4^+^ T-cell frequencies specific for RBD variants in T1 (n = 27) and T4 (n = 33). The Friedman test followed by the Dunn multiple comparison test was performed, and no significant differences were observed (p > 0.05). (B) Correlation between S- or RBD variant-specific CD4^+^ T cell frequencies and anti-RBD IgG titers. The Spearman rank correlation coefficient was used for statistical analysis. The lines in the figures represent the best fit curve of simple linear regression analyses. Red characters indicate R ≥ 0.4 with statistical significance (∗∗p < 0.01).

We also determined CD4^+^ T cell frequencies specific for RBD using a set of overlapping peptides covering RBD. Because S-specific CD4^+^ T cell responses correlated weakly with anti-RBD IgG titers at T1 and T4 (Figure 4), we examined the extent to which RBD-specific CD4^+^ T cell responses correlate with anti-RBD IgG titers at T1 (n = 27) and T4 (n = 33) (two samples at T1 could not be analyzed due to the limited sample availability). Since previous reports indicate mutations in RBD confer resistance to cellular immunity (Motozono et al., 2021), we also determined the frequencies of CD4^+^ T cells against RBD of wild type (WT), beta (containing K0417N, E0484K, N0501Y), and epsilon (containing L452R) and correlated the frequencies with anti-RBD IgG titers. As shown in Figure 5A, frequencies of CD4^+^ T cells responding to WT, beta, and epsilon RBD were similar. Interestingly, anti-RBD IgG titers correlated more strongly with the frequency of CD4^+^ T cells responding to WT-RBD than those to the entire S, beta-RBD, and epsilon RBD (Figure 5B).

### T cell subsets associated with disease severity

**Figure 6:**
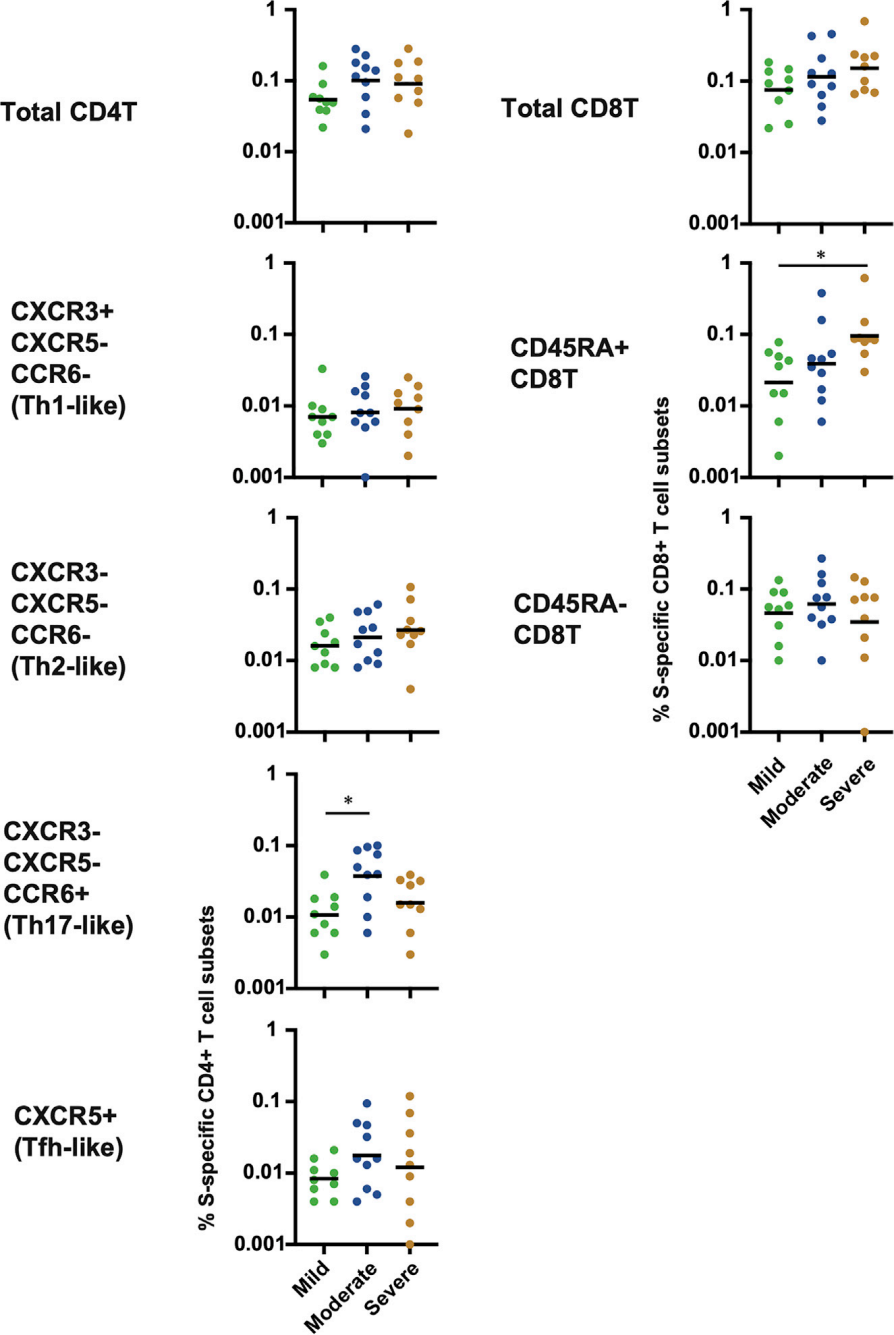
Comparison of S-specific CD4^+^ and CD8^+^ T cell frequencies between severities in T1 Significant differences (∗p < 0.05) were determined by the Kruskal-Wallis test followed by the Dunn multiple comparison test.

While previous reports suggested that SARS-CoV-2-specific T cell responses were inversely correlated with disease severity during disease progression (Mathew et al., 2020; Moderbacher et al., 2020), we did not observe significant differences in the frequencies of SARS-CoV-2-specific CD4^+^ and CD8^+^ T cells at the early memory phase in our cohort (Figures 2A and S2A). We then examined the association between disease severity and individual T cell subsets. CD4^+^ T cells were divided into Th1-, Th2- Th17-, and Tfh-like cells, while CD8^+^ T cells were divided into CD45RA^+^ and CD45RA^-^ subsets. As shown in Figure 6, the frequencies of S-specific CD45RA^+^ CD8^+^ T cells were significantly higher in severe cases than in mild cases. There was no correlation between S-specific CD45RA^+^ CD8^+^ T cells and subjects’ age. CD45RA^+^ CD8^+^ T cells represent not only effector T cells and terminally differentiated effector memory T cells (CD45RA^+^ CCR7-) but also stem cell-like memory (Tscm) T cells (CD45RA^+^ CCR7^+^) (Jung et al., 2021). To distinguish these alternatives, we analyzed the frequencies of SARS-CoV-2 S-specific CD45RA^+^ CD8^+^ T cells in PBMCs collected later than 8 months after the symptom onset (T4). As shown in Figure S6A, there was no significant difference between mild and severe cases at T4, indicating that the higher frequency of CD45RA^+^ CD8^+^ T cells in severe cases at T1 was most likely attributed to the induction of short-lived T cells rather than Tscm. Collectively, these results indicate that effector or terminally differentiated effector memory CD8^+^ T cells may contribute to the pathogenesis of COVID-19 in the convalescent phase.

## Discussion

Little information was available about factors that affect memory T cell longevity. In addition, the relationship between T cell responses and antibody titers after SARS-CoV-2 infection remains incompletely understood. Here, we analyzed T cell and antibody responses of a unique cohort in which similar numbers of mild (n = 15), moderate (n = 10), and severe (n = 13) COVID-19 cases were enrolled. In most cases, both plasma and peripheral blood samples were available at multiple time points, spanning approximately 1 year after COVID-19 infection. The sample availability at multiple time points enabled us to calculate the half-life of T cell and antibody responses without being affected by differences between individuals. The results revealed differential longevity among memory CD4^+^ T cell subsets. The study also identifies distinct CD4^+^ T cell subsets that are associated with anti-RBD IgG titers. Interestingly, anti-RBD IgG titers were more closely correlated with CD4^+^ T cell responses to RBD than to the entire S region during the memory phase.

Immune memory induced by primary infection or vaccination provides the foundation of protective immunity from subsequent infection. While sterilizing immunity can only be achieved by pre-existing neutralizing antibodies, T cells are thought to prevent the onset of symptomatic or severe disease. Detailed information on T cell durability is urgently needed because VOCs that can evade antibody neutralization are rapidly emerging. It has been difficult to compare the durability between SARS-CoV-2-specific antibodies and T cells, because longitudinal data with at least three time points per subject are rarely available and immune responses to SARS-CoV-2 infections are notably heterogeneous (Bert et al., 2020; Grifoni et al., 2020; Moderbacher et al., 2020; Sekine et al., 2020). As expected, SARS-CoV-2-specific T cell responses are more durable than antibody responses, reminiscent of SARS-CoV-1 and Middle East respiratory syndrome coronavirus (MERS-CoV) infection in which T cell responses persisted for 10 years even when antibody responses became undetectable in the same subjects (Tang et al., 2011; Zhao et al., 2017).

Interestingly, we found CCR6^+^ T cells were more stable than other subsets, suggesting differential decay kinetics among memory T cell subsets. Several studies have reported CCR6^+^ SARS-CoV-2-specific T cells in convalescent COVID-19 patients (Biasi et al., 2020; Juno et al., 2020; Moderbacher et al., 2020), but their durability was unknown. CCR6 is a phenotypical marker of activated/memory T cells (Kondo et al., 2007) but is frequently used as a Th17 marker as well (Hirota et al., 2007; Singh et al., 2008). Because CCR6^+^ SARS-CoV-2-specific CD4^+^ T cells in peripheral blood express little or no interleukin 17a (Braun et al., 2020; Grifoni et al., 2020; Rodda et al., 2021), CCR6 expression presumably reflects migration to the lung. Indeed, the expressions of CCR6 and its sole ligand, CCL20, were both enriched in the lungs of mechanically ventilated COVID-19 patients (Saris et al., 2021). In this regard, it is puzzling that CCR6^+^ CD4^+^ T cells persisted longer than other T cell subsets as effector memory T cells are supposedly short lived compared with central memory counterparts (Geginat et al., 2001; Pepper and Jenkins, 2011; Sallusto et al., 1999). Considering the documented SARS-CoV-2 persistence for an extended period (Gaebler et al., 2021), the longer half-life of CCR6^+^ CD4^+^ T cells may reflect the continuous generation of short-lived effector memory T cells. It should also be noted that we have only analyzed SARS-CoV-2-specific T cell subsets in peripheral blood. Therefore, other subsets may simply reside in lymphoid tissues, which are preferred by long-lived memory T cells (Geginat et al., 2001; Sallusto et al., 1999). In either case, given the reported nature of CCR6^+^ memory T cells, they should provide the first line of defense against reinfection. Further studies are required to determine the exact role of CCR6^+^ memory T cells upon reinfection and the mechanistic basis of differential memory maintenance among T cell populations.

Intriguingly, information about the correlation between SARS-CoV-2-specific antibody and T cell responses is quite limited. Because Tfh cells are critical for the generation of neutralizing antibodies and long-lived humoral immunity in most contexts (Crotty, 2015; Morita et al., 2011; Ueno et al., 2015), several studies have examined Tfh responses against SARS-CoV-2, in particular, specific for S-protein (Braun et al., 2020; Dan et al., 2021; Grifoni et al., 2020; Meckiff et al., 2020; Moderbacher et al., 2020). Although SARS-CoV-2-specific cTfh cell frequencies were shown to correlate with reduced disease severity (Juno et al., 2020; Moderbacher et al., 2020), their correlation with S-specific antibody titers was equivocal. Boppana et al. demonstrated that cTfh frequencies were associated with neutralizing antibody titers during the early convalescent phase but did not analyze other T cell subsets or later time points (Boppana et al., 2021). Extending the previous findings, our data indicate that the frequencies of Tfh cells remained to correlate with antibody titers up to 8 months after COVID-19 infection. It is unclear whether the T cell-B cell interaction continuously occurs for such an extended period. Notably, however, we and others have recently shown that the potency and breadth of neutralization antibodies continuously improve long after recovery from COVID-19 (Moriyama et al., 2021; Muecksch et al., 2021; Wang et al., 2021), consistent with sustained germinal center responses and antigen persistence (Poon et al., 2021).

We do not know the basis of which CD4^+^ T cell responses specific for the RBD region are most closely associated with RBD-specific antibody titers. A current immunological model postulates that antigen-engaged B cells must encounter cognate antigen-specific CD4^+^ T cells to differentiate into antibody-secreting plasma cells (Sette et al., 2008). This scenario is insufficient to explain why anti-RBD IgG titers were more closely correlated with RBD-specific CD4^+^ T cells than with S-specific CD4^+^ T cells, because RBD is contained in the S protein in its entirety and S-specific CD4^+^ T cells should be “cognate” T cells for RBD-specific B cells. It is possible that the relationship between S-specific CD4^+^ T cells and RBD-specific antibody titers is weakened by CD4^+^ T cells that cross-react with seasonal coronaviruses (Bert et al., 2020; Lineburg et al., 2021; Loyal et al., 2021). Because the amino acid sequence of RBD is relatively unique compared to seasonal coronaviruses, these cross-reactive S-specific CD4^+^ T cells may not interact with RBD-specific B cells as effectively as SARS-CoV-2 RBD-specific B cells.

Finally, the study revealed a potential involvement of CD45RA^+^ CD8^+^ T cells in COVID-19 pathogenesis. CD45RA^+^ CD8^+^ T cells could be CCR7^+^ CD45RA^+^ stem cell-like memory CD8^+^ T cells (Tscm), which were shown to be induced after SARS-CoV-2 infection by Jung et al. (2021). However, disease severity was presumably correlated with relatively short-lived, CCR7^-^ CD45RA^+^ T cells that are either effector T cells (Te) or terminally differentiated effector memory T cells (Temra), because the frequencies of CD45RA^+^ CD8^+^ T cells at T4 were similar between mild, moderate, and severe patients.

### Limitations of study

Limitations in our study include a relatively small number of subjects. This was necessary because we selected subjects who contributed blood samples at multiple time points for approximately 1 year. As the COVID-19 vaccine program progress worldwide, natural COVID-19 infections become rarer. Another limitation is insufficient knowledge on the protection level that different memory T cell subsets confer against reinfection. While sterile protection is achieved by neutralizing antibodies, memory T cells are expected to contribute to clearing the virus and regulating disease severity. Finally, memory T cell analyses in this study are restricted to circulating T cells. Memory T cells in the upper respiratory tract likely exhibit different phenotypes and may play a more important role upon reinfection. Nevertheless, the current study provides important information regarding the longevity and phenotypes of SARS-CoV-2-specific memory T cell responses, which may help establish a more efficient vaccine program to subdue the COVID-19 pandemic.

## STAR★Methods

### Key resources table

**Table:** 

REAGENT or RESOURCE	SOURCE	IDENTIFIER
**Antibodies**		

COVA1-18	Brouwer et al., 2020	MT599837.1, MT599921.1
SKOT-9	Ohnishi et al., 2005	N/A
HRP-conjugated goat anti-human IgG	Southern Biotech	Cat# 2040-05; RRID: AB_2795644
FcR Blocking Reagent, human	Miltenyi Biotec	Cat# 130-059-901; RRID: AB_22892112
PerCP anti-human CD3 Antibody (clone UCHT1)	BioLegend	Cat# 300428; RRID: AB_893298
Brilliant Violet 605™ anti-human CD4 Antibody (clone OKT4)	BioLegend	Cat# 317438; RRID: AB_11218995
Alexa Fluor® 700 anti-human CD8a Antibody (clone RPA-T8)	BioLegend	Cat# 301028; RRID: AB_493745
APC/Cyanine7 anti-human CD45RA Antibody (clone HI100)	BioLegend	Cat# 304128; RRID: AB_10708880
FITC anti-human CD69 Antibody (clone FN50)	BioLegend	Cat# 310904; RRID: AB_314839
APC anti-human CD137 (4-1BB) Antibody (clone 4B4-1)	BioLegend	Cat# 309810; RRID: AB_830672
BV786 Mouse Anti-Human CD183 (clone 1C6/CXCR3)	BD Biosciences	Cat# 741005; RRID: AB_2740628
BV421 Rat Anti-Human CXCR5 (CD185) (clone RF8B2)	BD Biosciences	Cat# 562747; RRID: AB_2737766
Brilliant Violet 650™ anti-human CD196 (CCR6) Antibody (clone G034E3)	BioLegend	Cat# 353426; RRID: AB_2563869

**Biological samples**		

SARS-CoV-2-infected convalescent patient blood sample	Tokyo Shinagawa Hospital and Tokyo Center Clinic	N/A

**Chemicals, peptides, and recombinant proteins**		

Vacutainer CPT tube	BD Biosciences	362761
SARS-CoV-2 recombinant RBD protein	In-house, Moriyama et al. (2021)	MN994467
SARS-CoV-2 recombinant N protein	In-house, this paper	MN994467
TALON® Metal Affinity Resin	Clontech	635653
Ni-NTA agarose	Qiagen	30230
Bovine serum albumin	Sigma-Aldrich	A2153
Tween-20	Fujifilm Wako Pure Chemicals	167-11515
Can Get Signal Immunoreaction Enhancer Solution 2	TOYOBO	NKB-301
OPD substrate	Sigma-Aldrich	P8287
RPMI-1640 with L-glutamine and phenol red	Fujifilm Wako Pure Chemicals	189-02025
Penicillin/streptomycin	Thermo Fisher Scientific	15140-122
GlutaMAX Supplement	Thermo Fisher Scientific	35050061
PepMix™ SARS-CoV-2 (Spike Glycoprotein)	JPT Peptide Technologies GmbH	PM-WCPV-S-1
PepMix™ SARS-CoV-2 (NCAP)	JPT Peptide Technologies GmbH	PM-WCPV-NCAP-2
PepMix™ SARS-CoV-2 (S-RBD)	JPT Peptide Technologies GmbH	PM-WCPV-S-RBD-1
PepMix™ SARS-CoV-2 (S-RBD B.1.351)	JPT Peptide Technologies GmbH	PM-SARS2-RBDMUT02-1
PepMix™ SARS-CoV-2 (S-RBD B.1.429/Epsilon)	JPT Peptide Technologies GmbH	PM-SARS2-RBDMUT04-1
Dimethyl sulfoxide	Sigma-Aldrich	D2650
LIVE/DEAD Fixable Aqua Dead Cell Stain Kit	Thermo Fisher Scientific	L34965

**Critical commercial assays**		

Elecsys Anti-SARS-CoV-2	Roche Diagnostics	518316181
Expi293 expression system	Thermo Fisher Scientific	A29133

**Experimental models: Cell lines**		

Expi293F™ Cells	Thermo Fisher Scientific	Cat# A14527; RRID: CVCL_D615

**Software and algorithms**		

FlowJo	BD Biosciences	N/A
Prism 9	GraphPad	N/A

### Resource availability

#### Lead contact

Further information and requests for resources and reagents should be directed to and will be fulfilled by the lead contact, Masanori Isogawa (nisogawa@niid.go.jp).

#### Material availability

In-house materials are available from the lead contact upon request with a completed Material Transfer Agreement.

### Experimental model and subject details

#### Study design

The aim of this study was to analyze the relationship between SARS-CoV-2 specific T cell responses, antibody titers, and disease severity over an extended period. Samples were acquired from 15 mild, 10 moderate, and 13 severe cases for up to 14 months after symptom onset. Most subjects (28 of 38 subjects) contributed blood samples four times, allowing us to determine the half-lives of T cells and antibodies without being affected by subject-to-subject variations. We also divided SARS-CoV-2 specific CD4^+^ T cells into Th1-, Th2-, Th17-, and Tfh-like subsets based on chemokine receptors expression to determine whether any particular T cell subsets are associated with T cell longevity, antibody titers, and disease severity.

#### Ethics

This study protocol was approved by the National Institute of Infectious Diseases Ethics Review Board for Human Subjects (permit numbers: 1231 and 1299). All participants provided written informed consent in accordance with the Declaration of Helsinki prior to enrollment.

#### Human subjects

SARS-CoV-2-infected individuals (confirmed using SARS-CoV-2 PCR on nasopharyngeal swab samples upon admission) were enrolled at Tokyo Shinagawa Hospital and Tokyo Center Clinic. Blood samples were collected longitudinally (2–4 time points) from a total of 38 convalescent individuals between May 2020 and July 2021. All convalescent individuals were not re-infected with SARS-CoV-2 and were not subjected to any COVID-19 vaccine during the course of sampling. Subjects were grouped into mild (n = 15), moderate (n = 10), and severe (n = 13) on the basis of severity (mild, no pneumonia; moderate, 93% < oxygen saturation [SpO2] < 96%; severe, SpO2 ≤ 93%). One asymptomatic individual was included in the mild group, and the day of first positive SARS-CoV-2 PCR was used in place of the day of symptom onset. The day of symptom onset in all individuals was between March and July 2020, prior to the initial detection of SARS-CoV-2 VOC in Japan (December 2020; hCoV-19/Japan/IC-0446/2020). All study participants were ≥18 years old. The study cohort comprised 52.6% female (F) and 47.4% male (M) adults (60.0% F and 40.0% M in mild, 40.0% F and 60.0% M in moderate, and 53.8% F and 46.2% M in severe cases). The influence of gender on the results of the study was not explicitly measured.

### Method details

#### Preparation of PBMCs and plasma

Blood samples were collected in Vacutainer CPT tubes (BD Biosciences) and centrifuged at 1800 × *g* for 20 min at room temperature. PBMCs and plasma were collected and centrifuged at 300 × *g* for 15 min at room temperature. For plasma, the supernatant was harvested, centrifuged at 800 × *g* for 15 min at room temperature, and stored at −80°C until use. For PBMCs, the cell pellet was washed twice with PBS and cryopreserved at −135°C until use.

#### Recombinant proteins

The human codon-optimized nucleotide sequence encoding for the RBD and N protein of the CoV2 isolate (GenBank: MN994467) was synthesized commercially (Eurofins Genomics). The N protein (amino acids 1–419), along with the signal peptide (amino acids 1–24; MPMGSLQPLATLYLLGMLVASCLG), a histidine-tag, and an avi-tag was cloned into the mammalian expression vector pCMV. RBD (amino acids: 331–529) with the signal peptide (amino acids 1–20; MIHSVFLLMFLLTPTESYVD) and a C-terminal histidine tag was cloned into the mammalian expression vector pCAGGS (Moriyama et al., 2021). Recombinant proteins were produced using Expi293F cells according to the manufacturer’s instruction (Thermo Fisher Scientific). The supernatant from transfected cells was harvested on day 5 post-transfection, and recombinant proteins were purified using TALON Metal Affinity Resin (Clontech) or Ni-NTA agarose (Qiagen).

#### ELISA

F96 Maxisorp Nunc-Immuno plates (Thermo Fisher Scientific) were coated with 2 mg/mL of recombinant RBD or N proteins overnight at 4°C. After washing with PBS, the plates were blocked with 1% BSA in PBS for 1.5 h at room temperature. Heat-inactivated plasma and reference monoclonal antibodies (mAbs) against RBD (COVA1-18) (Brouwer et al., 2020) or against N (SKOT-9) (Ohnishi et al., 2005) were serially-diluted in PBS containing 1% BSA and 0.05% Tween 20 (eight 4-fold serial dilutions starting at 1:20 dilution for plasma, or eight 4-fold serial dilutions starting at a 1 mg/mL for mAbs), and then incubated overnight at 4°C. The following day, the plates were washed with PBS containing 0.05% Tween 20. HPR-conjugated goat anti-human IgG (Southern Biotech) was diluted in Can Get Signal Immunoreaction Enhancer Solution 2 (TOYOBO) and incubated for 1.5 h at room temperature. HRP-activity was visualized by the addition of OPD substrate (Sigma-Aldrich), and OD490 was measured using iMark microplate reader (Bio-Rad).

#### Activation-induced markers (AIM) T cell assay

PBMCs were suspended in R10 medium (RPMI-1640 medium supplemented with 10% fetal bovine serum, 100 μg/mL penicillin, 100 μg/mL streptomycin, and 1% GlutaMAX Supplement [Thermo Fisher Scientific]) and incubated at 0.5–1.5 × 10^6^ cells/well in 96-well U-bottom plates with the relevant peptides at 37°C for 16 h in a 5% CO_2_ incubator. All of the overlapping (15 oligomers with 11 amino acids overlap) peptide pools spanning the spike glycoprotein of wild type (WT) strain (PM-WCPV-S-1), nucleoprotein of WT strain (PM-WCPV-NCAP-2), RBD of WT strain (PM-WCPV-S-RBD-1), RBD of B.1.351 strain (PM-SARS2-RBDMUT02-1), and RBD of B.1.429 strain (PM-SARS2-RBDMUT04-1) were purchased from JPT Peptide Technologies, reconstituted with dimethyl sulfoxide (DMSO), and used at a final concentration of 1 μg/mL. Stimulation with solvent (DMSO) alone was performed as a mock negative control. After incubation, cells were washed with staining buffer (PBS supplemented with 2% fetal bovine serum and 0.01% NaN3) and then stained for 1 h on ice with LIVE/DEAD Fixable Aqua Dead Cell Stain Kit (Thermo Fisher Scientific) and the following antibodies: the FcR Blocking Reagent, human (1:100, Miltenyi Biotec), CD3-PerCP (UCHT1, 1:100, BioLegend), CD4-BV605 (OKT4, 1:100, BioLegend), CD8-AF700 (RPA-T8, 1:200, BioLegend), CD45RA-APC-Cy7 (HI100, 1:250, BioLegend), CD69-FITC (FN50, 1:100, BioLegend), CD137-APC (4B4-1, 1:100, BioLegend), CD183/CXCR3-BV786 (1C6/CXCR3, 1:17, BD Biosciences), CD185/CXCR5-BV421 (RF8B2, 1:20, BD Biosciences), and CD196/CCR6-BV650 (G034E3, 1:25, BioLegend). After staining, cells were washed twice with staining buffer and then subjected to flow cytometry using a FACS Aria III cytometer (BD Biosciences). Data were saved as FCS files and analyzed using FlowJo software (v. 10.8.0, BD Biosciences). A representative gating strategy is shown in Figure S1. CD69 and CD137 were used as activation-induced markers, and antigen-specific CD69^+^CD137^+^ T cells were measured as mock-subtracted data. The percentage of antigen-specific T-cells is represented as the frequency in total CD4^+^ or CD8^+^ T cells, and 0.001% was set as the detection limit on the basis of the cell number acquired.

### Quantification and statistical analysis

Statistical analysis was performed using Prism 9 (GraphPad). Statistical test for each analysis is described in each legend. Statistical significance was defined as p < 0.05.

## Data Availability

All data reported will be shared by the lead contact upon request. This paper does not report the original code. Additional information required to analyze the data reported in this paper is available from the lead contact upon request.
