# Supplementary material for: SARS-CoV-2-specific CD4+ T cell longevity correlates with Th17-like phenotype
Source: iScience. 2022 Aug 17;25(9):104959. doi: 10.1016/j.isci.2022.104959 (PMC9384329; doi:10.1016/j.isci.2022.104959)
Supplement: Document S1. Figures S1–S6 [file mmc1.pdf]

iScience, Volume *25*

Supplemental information

SARS-CoV-2-specific $CD4^+$ T cell longevity correlates with Th17-like phenotype

Kazutaka Terahara, Takashi Sato, Yu Adachi, Keisuke Tonouchi, Taishi Onodera, Saya Moriyama, Lin Sun, Tomohiro Takano, Ayae Nishiyama, Ai Kawana-Tachikawa, Tetsuro Matano, Takayuki Matsumura, Masaharu Shinkai, Masanori Isogawa, and Yoshimasa Takahashi

Supplementary Information

Gating strategy for each T-cell subset

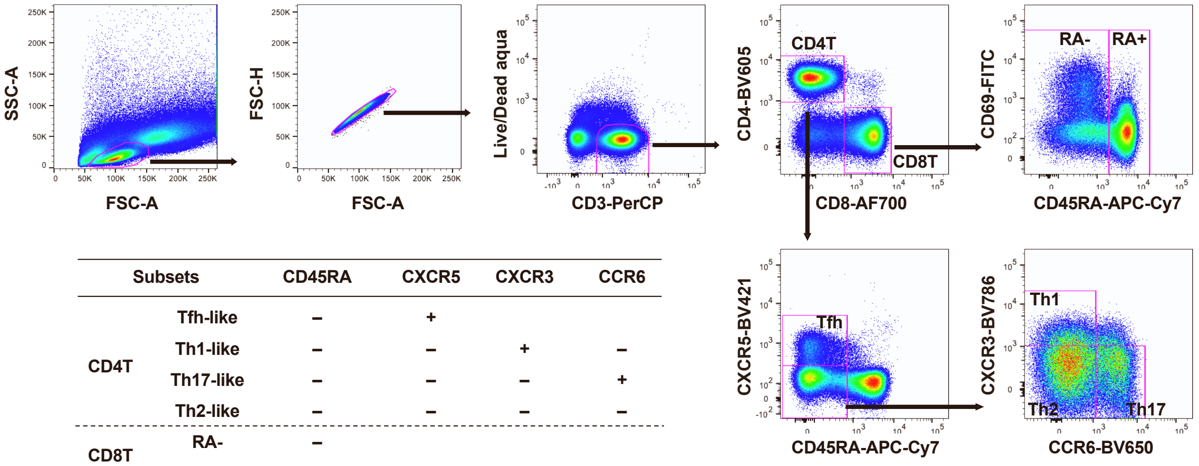

	Subsets	CD45RA	CXCR5	CXCR3	CCR6
CD4T	Tfh-like	–	+		
	Th1-like	–	–	+	–
	Th17-like	–	–	–	+
	Th2-like	–	–	–	–
CD8T	RA-	–			
	RA+	+			

Detecting AIM+ T-cell subsets
(exp. Mi-5 T1)

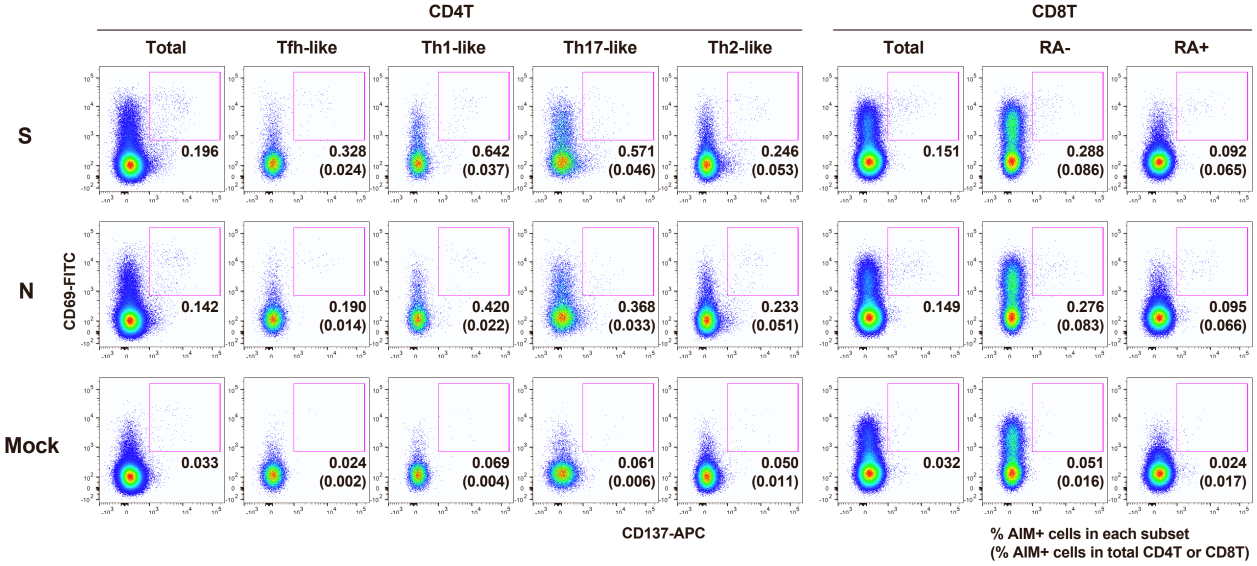

Figure S1. Representative gating strategies for SARS-CoV-2-specific CD4^{+} and CD8^{+} T cell subsets by AIM assay, related to STAR Methods.

S, N, and Mock indicate the stimulation with spike and nucleocapsid peptide pools and DMSO, respectively. CD69 and CD137 were used as activation-induced markers (AIM).

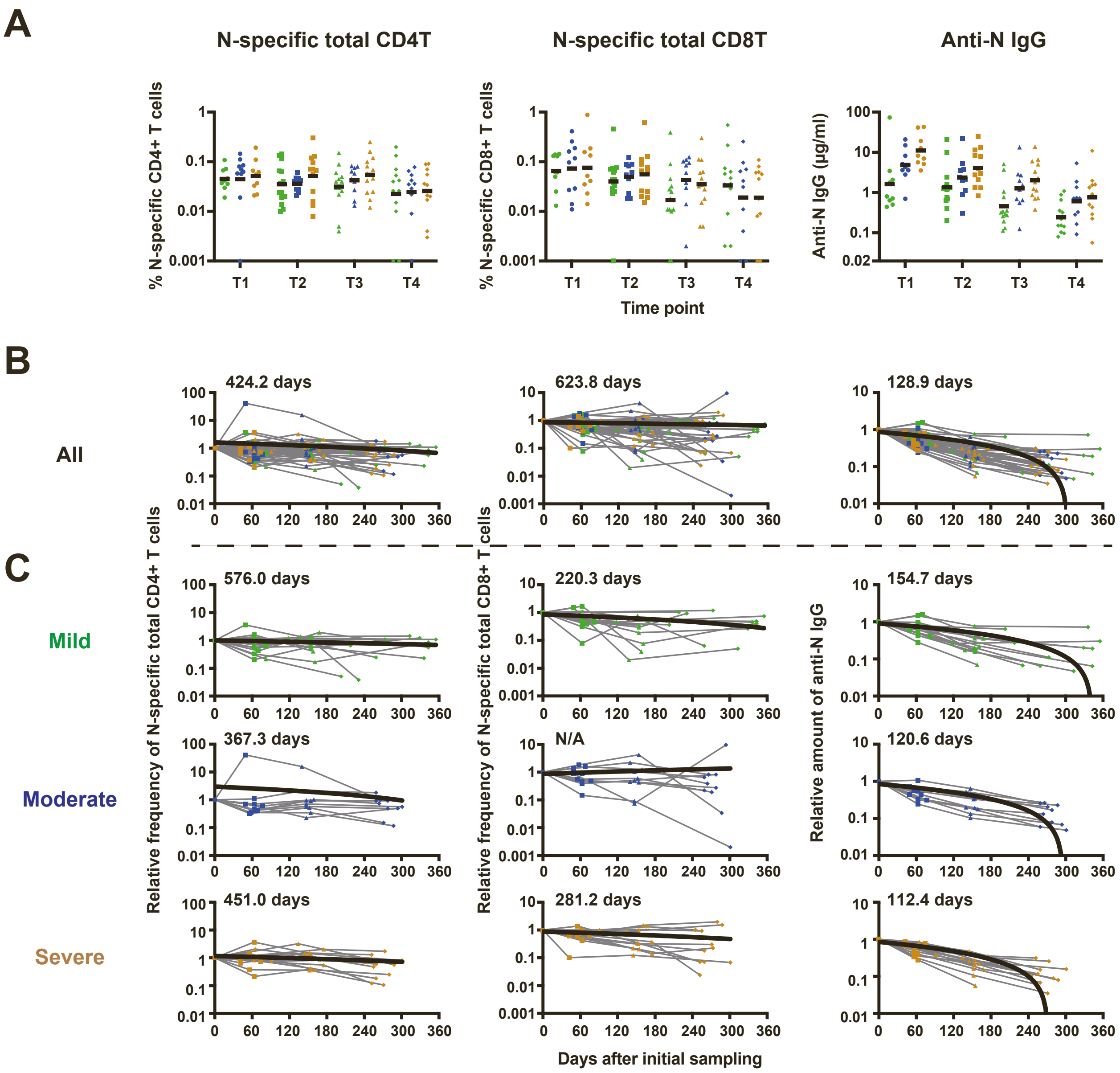

Figure S2. Kinetics of N-specific T cell frequencies and anti-N IgG titers by severity, related to Figure 2.

(A) N-specific CD4 T-cell frequencies in each time point. The mixed effects model followed by the Tukey's multiple comparison test was performed, and no significant differences were observed ($P > 0.05$) between the severity cases in each time point. (B and C) Half-life for N-specific T-cell frequencies and anti-N IgG titers in all subjects (B) and subjects divided into three groups based on disease severity (C) was calculated by the one-phase decay model, in which the initial sampling day was set as Day 0.

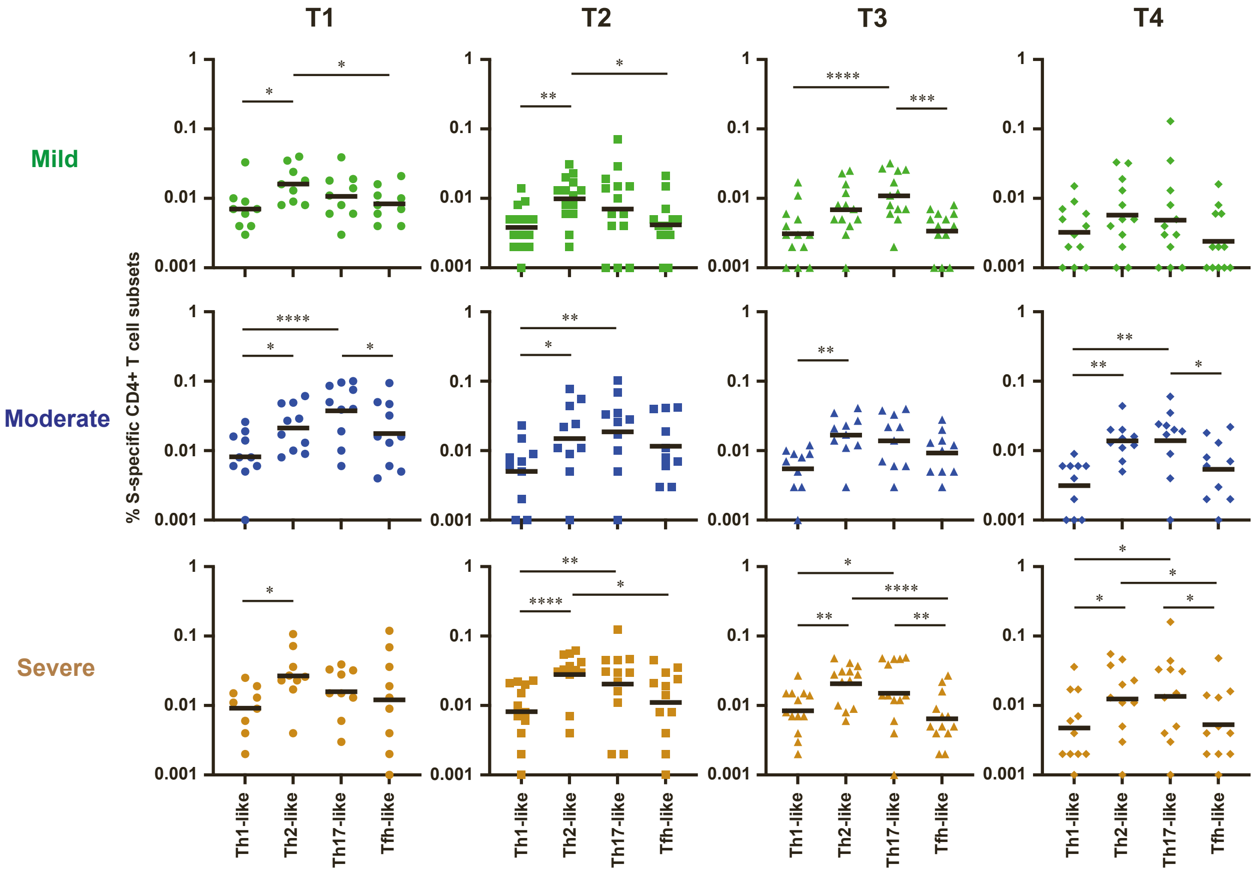

Figure S3. S-specific CD4$^+$ T cell frequencies at subset levels by severity, related to Figure 3.

Significant differences (*$P < 0.05$, **$P < 0.01$, ***$P < 0.001$, ****$P < 0.0001$) were determined by the Friedman test followed by the Dunn's multiple comparison test.

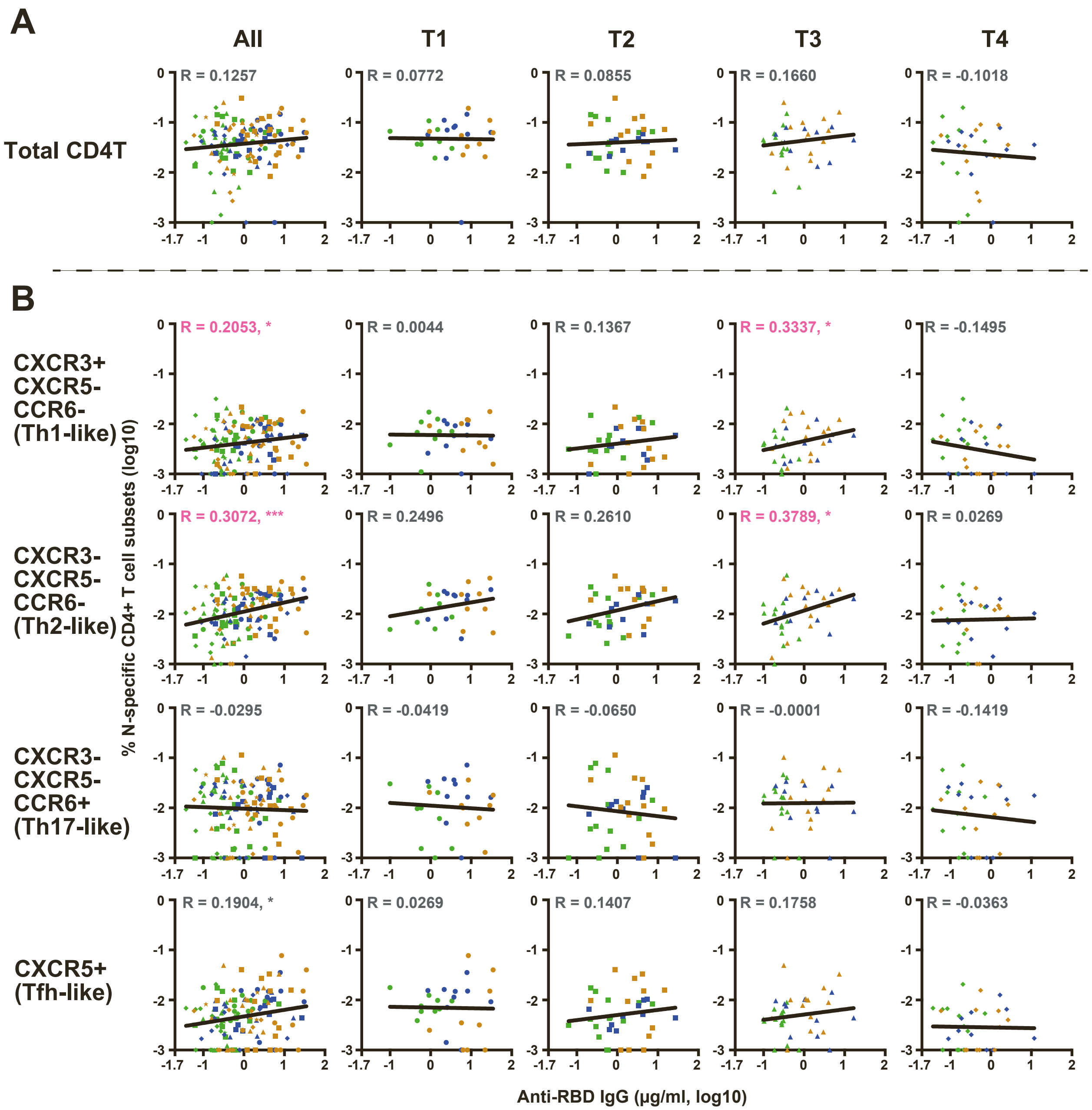

Figure S4. Correlation between N-specific CD4^{+} T-cell frequencies and anti-RBD IgG titers by time points, related to Figure 4.

The Spearman's rank correlation coefficient was used for statical analysis. Pink characters indicate R < 0.4, respectively, with statistical significance (**P* < 0.05, ****P* < 0.001).

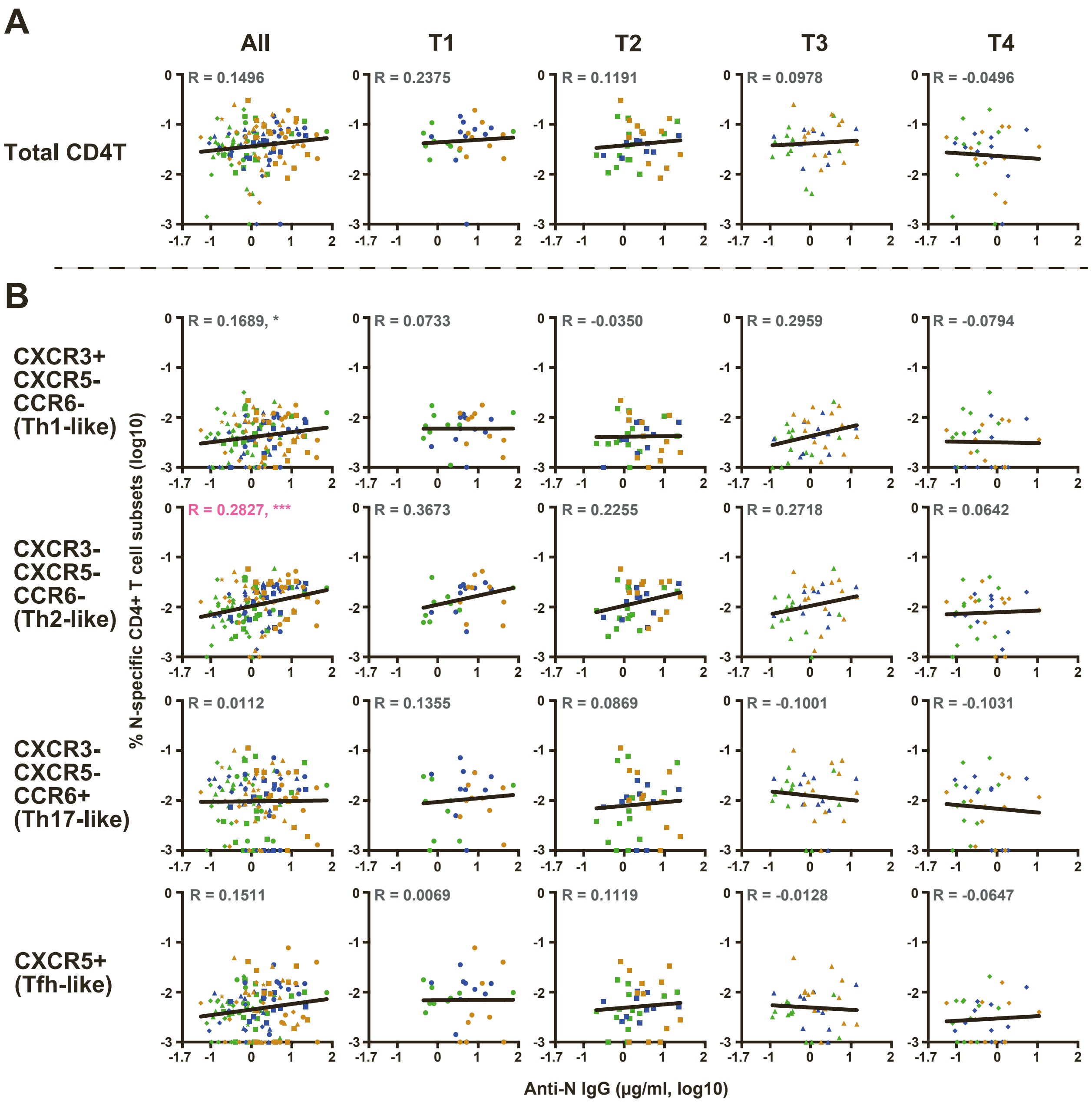

Figure S5. Correlation between N-specific CD4^{+} T-cell frequencies and anti-N IgG titers by time points, related to Figure 4.

The Spearman's rank correlation coefficient was used for statical analysis. Pink characters indicate R < 0.4, respectively, with statistical significance (****P* < 0.001).

[ここに入力]

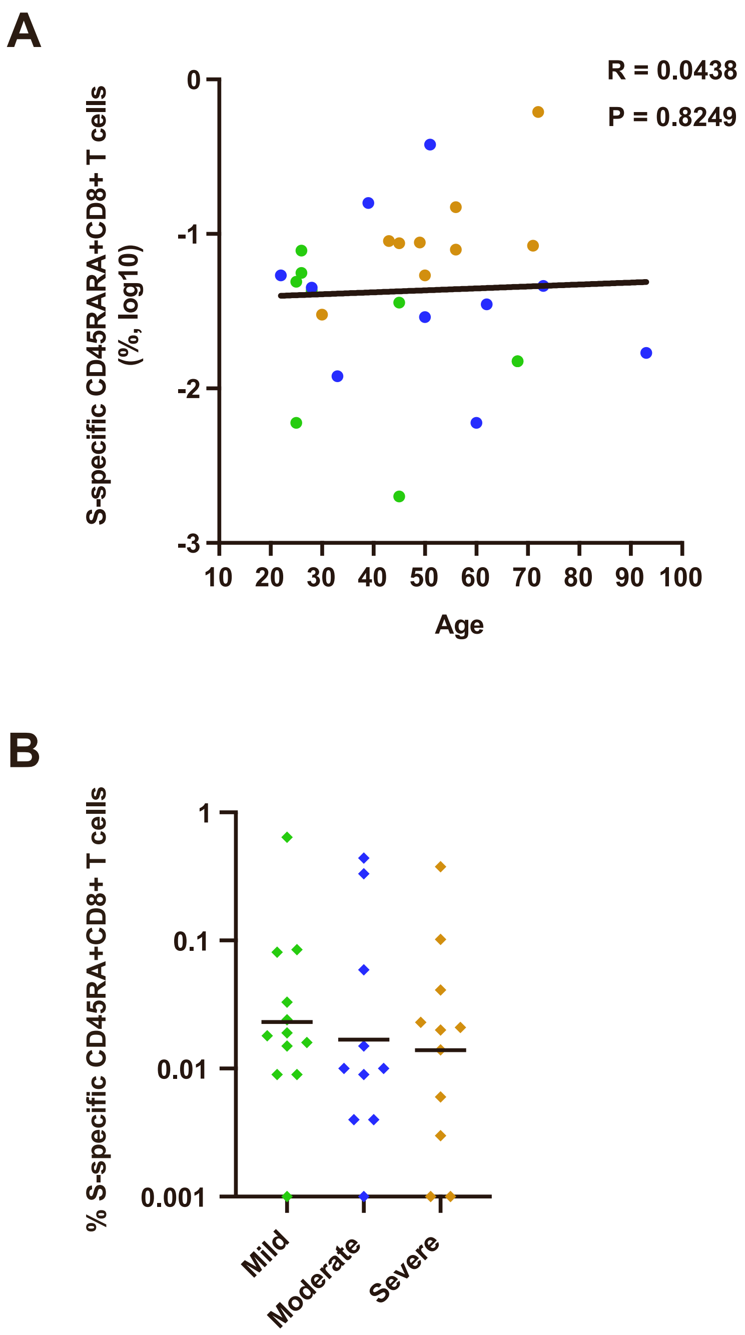

Figure S6. Characteristics of S-specific CD45RA+ CD8+ T cells, related to Figure 6.

(A) Correlation between S-specific CD45RA+ CD8+ T cells and ages in T1. The Spearman's rank correlation coefficient was performed for statistical analysis and no significant correlation was determined (R = 0.0438, P = 0.8249). (B) S-specific CD45RA+ CD8 T-cell frequencies inT4. The Kruskal-Wallis test followed by the Dunn's multiple comparison test was performed for statistical analysis and no significant differences were determined.

[ここに入力]
